# Unique surface and internal structure of struvite crystals formed by *Proteus mirabilis*

**DOI:** 10.1007/s00240-012-0501-3

**Published:** 2012-08-22

**Authors:** Jolanta Prywer, Agnieszka Torzewska, Tomasz Płociński

**Affiliations:** 1 Institute of Physics, Technical University of Lodz, ul. Wólczańska 219, 93-005 Lodz, Poland; 2 Department of Immunobiology of Bacteria, Institute of Microbiology and Immunobiology, University of Lodz, ul. Banacha 12/16, 90-237 Lodz, Poland; 3 Faculty of Materials Science and Engineering, Warsaw University of Technology, ul. Wołoska 141, 02-507 Warsaw, Poland

**Keywords:** Infectious urinary stones, Struvite, Crystal growth, *Proteus mirabilis*

## Abstract

Crystallization of struvite from artificial urine in the presence of *Proteus mirabilis* microorganisms depends strongly on pH value. At small value of pH, struvite yields crystals of coffin-like habit with very specific structure. The analysis using scanning electron microscopy shows that the crystals possess well-defined faces, but higher magnifications show very specific structuration as if the crystals were built from small three-dimensional subunits. The possible role of microorganisms in the formation of such a structuration is analyzed. At higher pH value, the crystals exhibit dendritic growth with main trunk and branches. Although the formation mechanism of the specific structuration as well as dendritic structures is unknown, the nature of forces for such an alignment is analyzed. The revealed porous internal structure of struvite is also analyzed. The investigations provide evidence for the importance of biological regulation in crystallization process.

## Introduction

Magnesium ammonium phosphate hexahydrate, MgNH_4_PO_4_·6H_2_O, known as struvite is an inorganic material whose crystallization is more and more wider investigated for several reasons. Struvite can be a problem in sewage and wastewater treatment as it precipitates easily on points with high turbulence such as elbows of pipe connections, valves, aeration assemblies and pump internal components exposed to wastewater [1, 2]. At these places, struvite may grow rapidly leading to system pipe clogging. On the other hand, struvite is a potential source of phosphorus, nitrogen and magnesium, and therefore it is the main compound recovered from wastewater and recycled as a useful P–N–Mg-containing fertilizer [3, 4].

The second and the most important reason for which struvite is widely investigated is the fact that it is the main component of so-called infectious urinary stones [5]. It is formed when the urinary tract is infected by urease positive microorganisms, mainly from *Proteus* sp. Urease, an enzyme produced by these microorganisms, splits urea, (NH_2_)_2_CO—a natural component of urine—into ammonia NH_3_ and carbon dioxide CO_2_. These products alkalinize urine. Under alkaline conditions, an increase in the concentration of the NH_4_^+^, CO_3_^2−^ and PO_4_^3−^ ions occurs. These ions, together with Mg^2+^ ions physiologically present in the urine, favor the crystallization of struvite. Struvite formation is usually associated with the precipitation of carbonate apatite [CA, Ca_10_(PO_4_)_6_CO_3_], because the Ca^2+^ ions are present in the urine. This component precipitates in an amorphous form. Struvite together with small amount of CA (up to 10 %) forms the so-called infectious stones. Since urinary stones are identified according to the predominant mineral present in their composition, these infectious stones are also called struvite stones. This kind of stones constitutes from 10 % [5] to 30 % [6, 7] of all urinary stones. The frequency of their occurrence depends on the country and the degree of its industrialization. They are found more frequently in economically developed countries. It seems that this must be attributed to the different lifestyles and dietary habits. Struvite stones may grow rapidly and, if not adequately treated, can develop into a large stone that fills the entire intra-renal collecting system. Patients with infectious stones left untreated have about a 50 % chance of losing a kidney [6, 8, 9].

In view of the above reasons, struvite crystallization has been increasingly investigated over the last years. Preliminary investigations demonstrate that struvite crystals grown in in vitro conditions in the presence of microorganisms have well-defined faces, but they show very specific structures and appear like built from small subunits [10, 11]. Moreover, gaps between small units from which the crystal is built may be identified, suggesting the porous nature of struvite. Porosity and small building units may suggest that struvite is mesocrystal [12].

In this paper, we analyze in detail the specific structuration of struvite, its morphology and habit modification and the role of microorganisms in this structuration. For this purpose, we performed sets of experiments of struvite crystallization from artificial urine. The crystallization process was induced by *P. mirabilis* to mimic the real urinary tract infection, which usually leads to urinary stone formation.

## Materials and methods

*Proteus mirabilis* strain was isolated from human urinary stone. Before the crystal growth experiment, bacteria were maintained on a slant of tryptic soy agar overnight at 37 °C and then suspended in artificial urine to the concentration of 5 × 10^5^ CFU/ml.

The artificial urine used during crystal growth experiments was made from the following components [13] (g/l): CaCl_2_·2H_2_O, 0.651; MgCl_2_·6H_2_O, 0.651; NaCl, 4.6; Na_2_SO_4_, 2.3; KH_2_PO_4_, 2.8; KCl, 1.6; NH_4_Cl, 1.0; sodium citrate, 0.65; sodium oxalate, 0.02; urea, 25.0; creatine, 1.1; and tryptic soy broth, 10.0. All chemicals were purchased from Sigma and were used without further purification. The tryptic soy broth was added to stimulate the bacterial growth, but not anticipated to interfere with the precipitation process and does not sequester constituent ions of the precipitating crystalline and amorphous phases.

Mineral components in the above artificial urine correspond to mean concentration found in 24 h period in normal human urine. Crystallization occurs after addition of the suspension of bacteria and incubation at 37 °C. The crystallization process occurs at conditions emulating the natural conditions existing in human body during the infection by *Proteus* sp. The experimental procedure is described in detail elsewhere [9, 14]. Control experiments without bacteria were performed using artificial urine of the same composition. In this case, the crystallization process occurs after consecutive addition of aqueous ammonia solution (1 M) to mimic urease activity under the control of pH value.

The pH of the solution of artificial urine was screened along the experiments using digital pH-meter (Elmetron CP-215). The initial pH of artificial urine was adjusted to a value of 5.8. During all the growth experiments, crystals samples were taken at regular intervals and observed by optical microscopy (Opta-Tech MN800) and scanning electron microscopy (SEM). The SEM micrographs were performed using a Vega 5135 MM—Tescan microscope. The internal structure of the investigated crystals was revealed with the aid of a focused ion beam (FIB) microscope (Hitachi FB-2100). In this system, a highly focused ion beam with beam diameter of 6 nm is applied. As the ion source liquid Ga^+^ ions are applied.

Crystals were identified as struvite by X-ray diffraction (XRD), using X’pert PRO MPD (PANanalytical) diffractometer. The Cu K_α_ radiation monochromatized by nickel filter was applied. The X’pert HighScore Plus (PANanalytical) software was used to analyze XRD patterns. The crystals for XRD and FIB examination were separated from bacteria suspension and CA precipitation in sedimentation process—the settling process proceeds more rapidly for larger-sized particles.

## Results and discussion

### Specific structuration of struvite

Struvite belongs to the non-centrosymmetric point group *mm2* of the orthorhombic system with the space group *Pmn2*_1_, with the lattice parameters [15]: *a* = 6.966(1) Å, *b* = 6.142(1) Å and *c* = 11.217(2) Å.

**Fig. 1:**
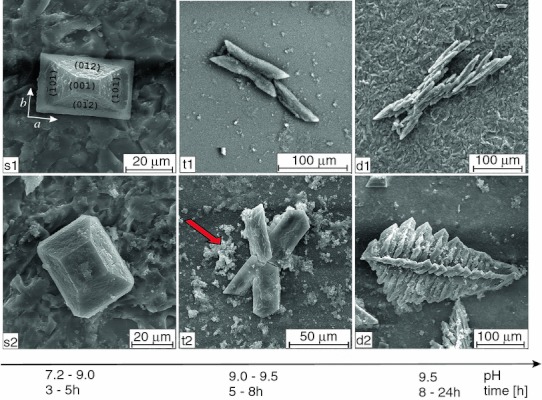
SEM micrographs illustrating the dependence of morphology and habits of struvite on pH of artificial urine; *left column*: single hemimorphic crystals obtained for small pH values; *middle column*: contact (*t1*) and penetration (*t2*) twins obtained for higher pH values; *right column*: X-shaped (*d1*) and fern-leaf (*d2*) dendrite crystals obtained for the highest values of pH; the image in* d1 panel* is reproduced from Ref. [10]; *arrow* indicates CA precipitation

In order to study the mineralization of struvite, we performed several sets of experiments of struvite crystallization from artificial urine with the presence of *P. mirabilis*. The results of our experiments are presented in Fig. 1. Crystal morphology and growth process strongly depends on pH (Fig. 1), which increases with time because of urease activity. First single crystals appear approximately after 3 h at pH 7.2. With time and with increase in pH, the amount of crystals is greater. The size of the largest crystals is about 60 μm along the *a* axis (the crystallographic directions are defined in Figs. 1, panel s1 and 6). The aspect ratio (AR) defined as the crystal length *l*_a_ along the *a* axis to the length *l*_b_ along the *b* axis varies from 1 to 2. The basic crystal morphology is coffin-like (Fig. 1, panels s1, s2) and it is very typical for crystals growing in living animal and human organisms [16, 17]. The coffin-like morphology is typical hemimorphic morphology which means that the two ends of a crystallographic *c* axis are not related by symmetry [18]. The detailed explanation of this issue and analysis of struvite morphology is presented by Prywer and Torzewska [10, 14]. When pH increases further, the habits of single crystals remain the same but the crystals very frequently form twins. Initially, the crystals show contact twining (Fig. 1, panel t1), and then contact twining turns into penetration twining (Fig. 1, panel t2). For highest value of pH 9.5, large dendritic branches and dendritic structures appear (Fig. 1, panels d1, d2). Such dendritic structures may easily be retained in the urinary tract and physically damage the epithelium of internal renal walls. The sites of damaged epithelium are places where the adhesion is greater. In those places, bacteria or nucleus of crystalline phase can settle easier than in other places. Therefore, the sites of damaged epithelium, and consequently, dendritic structures can facilitate the nucleation process and promote the crystallization of struvite. In all photographs presented in Fig. 1, one may notice amorphous deposition of CA (arrow) which precipitates, in a small amount, as described in “Introduction”.

**Fig. 2:**
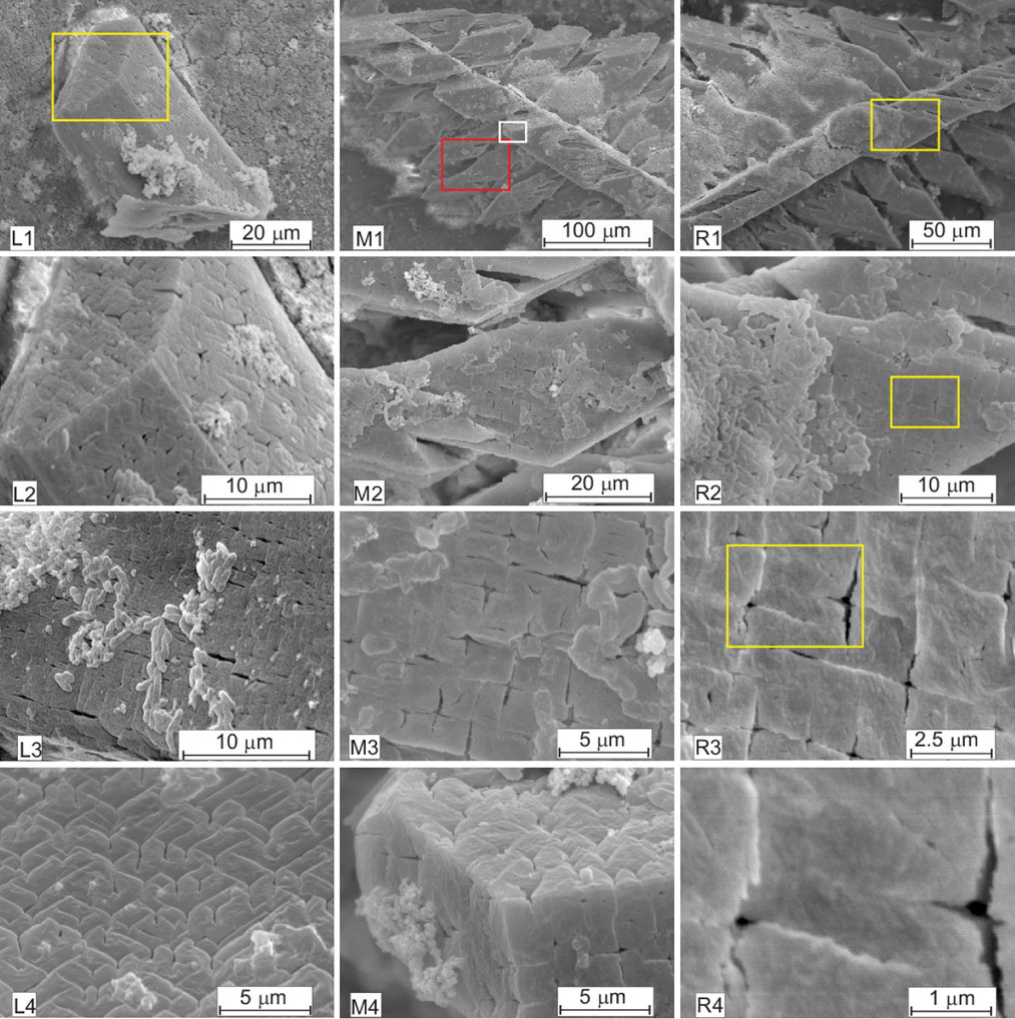
SEM micrographs of struvite crystals at higher magnification revealing specific structuration of their surfaces; *left column*: single hemimorphic morphology,* L2* is a higher magnification of *rectangle area* marked in* L1*; *middle* and *right columns*: dendrite-like structures;* M2* and* M4* are higher magnification of the *rectangle areas* marked in* M1* by colors *red* and *white*, respectively;* M3* is higher magnification of the *rectangle area* marked in* M2*;* R2*,* R3* and* R4* are higher magnification of the *rectangle areas* marked in* R1*,* R2* and* R3*, respectively

From the enlarged pictures (Fig. 2), one may note that the faces of single crystals are well defined (Fig. 2, panels L1, L2), but it seems that the crystal is constructed from small subunits with average size of 1–3 μm. The picture L2 shows the rectangle area marked in L1 at a higher magnification. Another single crystal with such structure is shown in Fig. 2, L3. The rod-like structures visible here are bacteria. The picture L4 (Fig. 2) shows similar structure of the $(00\bar{1})$ face (the bottom of the coffin-like habit) of struvite crystal. On the basis of Fig. 2, left column, gaps between small units from which the crystal is built may be identified, suggesting the porous nature of struvite. The size of the gaps is in the range from tens to hundreds of nanometres. The size of the largest gaps is about 500 nm. These results are consistent with the results presented in [9, 10, 19] and may suggest that struvite is a mesocrystal. However, predominantly, mesocrystals are aggregates of nanoparticles and therefore the results may suggest that our case is borderline case.

When pH value increases rapidly, the presence of single crystals vanishes progressively and the majority of the crystals are hopper and dendrite-like. In some cases, single, hopper and dendrite-like crystals have been observed coexisting. The hopper crystals correspond to an intermediate stage of evolution between the single and dendrite-like crystals. The evolution of dendrite-like crystals is mainly influenced by the rate of change in pH with minor influence of the value of pH. The absence of dendrite-like structures is a key indication that sample did not experience a rapid change in pH value. The virtual boundary separating these two kinds of crystals (single and dendrite-like) can be considered as the limit between the slow and rapid growth. This finding is consistent with the investigations presented in Ref. [20], in which the authors state that the morphology of crystals and their aggregates reflect the rate of crystallization and suggest the mechanism of stone formation [20]. The obtained twins and dendritic structures are illustrated in Fig. 1, middle and right columns, respectively. Dendrites are X-shaped (Fig. 1, panel d1) or they are composed of one trunk and branches symmetrically distributed on two sides of the trunk (Fig. 1, panel d2). All branches on one side of the trunk are parallel to each other and grow at an angle of ~77° with respect to the trunk. The branches are all located in the same plane. The magnified images (Fig. 2, middle and right columns) demonstrate that dendritic structures show the same characteristic structuration as in the case of single crystals. Picture M1 (Fig. 2) shows exemplifying dendritic structures. Pictures M2 and M4 show rectangle areas, red and white, respectively, marked in picture M1, at a higher magnification. Picture M3 is higher magnification of the rectangle area marked in M2. From these enlarged pictures, it may be observed clearly the characteristic pattern on the crystal surfaces. Picture M4 shows that this pattern is characteristic not only for a given surface but it is also characteristic for all surfaces. The right column of Fig. 2 (panels R1–R4) shows another example of dendritic structures with this characteristic structuration. Panels R2, R3 and R4 show rectangle areas marked in R1, R2 and R3 panels, respectively, at higher magnification. The example of this dendritic structure also shows characteristic pattern and demonstrates that the obtained structuration is not accidental.

**Fig. 3:**
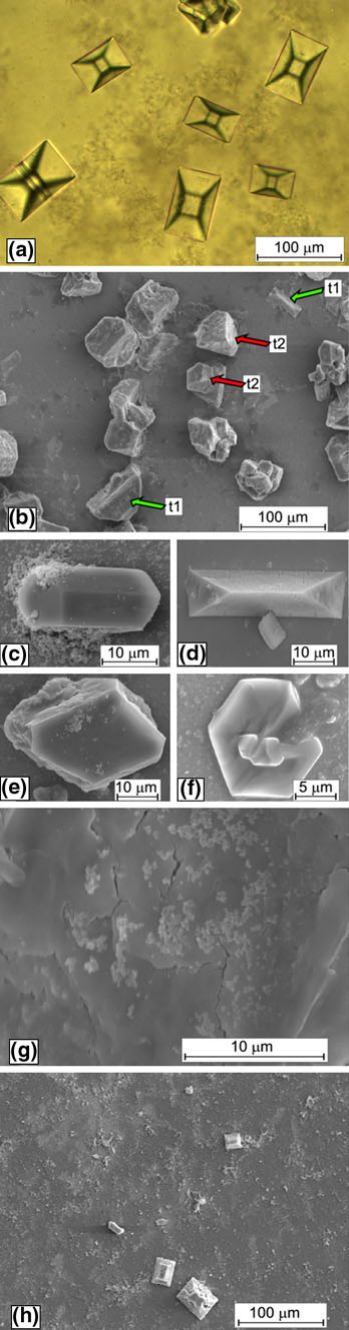
Struvite crystals obtained **a**–**f** in the control experiments without bacteria and **h** in the presence of bacteria; **g** struvite surface structure in the case of control experiment without bacteria;* t1* and* t2* in **b** indicate coffin-like and hexagonal habits, respectively

**Fig. 4:**
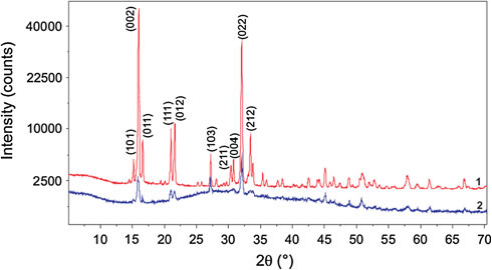
XRD patterns of struvite grown from artificial urine with the presence (curve *1*) and with the absence (curve *2*) of *P. mirabilis*

In order to understand the role of *P. mirabilis* in the formation of struvite structuration, we investigated the crystallization of struvite in the absence of bacteria. Such investigations are necessary to prove that SEM images show real structures instead of drying artifacts that result from sample preparation. The results are presented in Fig. 3. In the absence of bacteria, a relatively large number of crystals are formed (Fig. 3a) compared with the case of the presence of bacteria (Fig. 3h). In the case of absence of bacteria, the number of crystals is increased and so is their size—compare Fig. 3a, h. In most cases, the crystals are of coffin-like habits (Fig. 3a), but the crystals of other habits are also observed but rarely (Fig. 3b–f). In particular, some of the crystals take hexagonal habits (Fig. 3b, f). None of the crystals shows the structuration observed in the case of the presence of bacteria. Even at high magnification (Fig. 3g), we do not observe the characteristic structuration, instead we can see cracks which probably follow from the fact that the crystals were in the vacuum. To be sure that in both cases, we deal with struvite we have done the XRD analysis. Figure 4 shows XRD patterns of the obtained precipitation in both cases: in the presence (pattern denoted by 1) and in the absence of bacteria (pattern denoted by 2). These patterns reveal that the *d*-spacings (2θ value) are the same for the important lines and are comparable with the reference data. Therefore, the XRD patterns confirm that the precipitation is struvite in both cases. From these results, it can be concluded that *P. mirabilis* play a crucial role in directing the characteristic struvite structuration. It is suggested that the extracellular matrices of microorganisms mainly composed of proteins, and polysaccharides play crucial role in this structuration. This matter requires further research and analysis. Very similar pattern of struvite crystals was observed by Chen et al. [11]. In the case of investigations presented in Ref. [11], the crystals were grown in the presence of *P. mirabilis* in the appropriate solution which was not the solution of artificial urine. The observed surface pattern confirms that such a structuration is characteristic for the presence of bacteria.

**Fig. 5:**
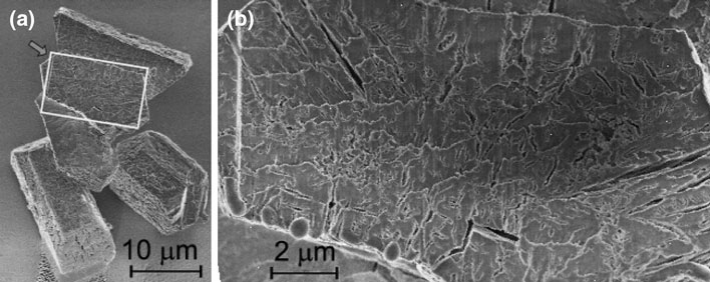
Struvite crystals grown in the presence of *P. mirabilis*, one of the crystal marked in **a** by an *arrow* is cut though with the aid of FIB system; **b** the higher magnification of the *rectangle area* marked in **a** revealing the porous internal structure of struvite with tubular pores

It is interesting to know whether this structuration is characteristic for surfaces only or for bulk structure and whether the big crystals are built from small subunits. In order to find out this matter, we have investigated the crystals with FIB microscope. The results of these investigations are presented in Fig. 5. Figure 5a presents an aggregate of few struvite crystals of coffin-like habit with characteristic structure. One of the crystals forming this aggregate, the one marked by the arrow, is cut though with the aid of FIB microscope. The crystal was cut approximately in the half of its thickness, i.e. about 10 μm from the external surface. Figure 5b presents the obtained cross-section at the higher magnification. The revealed internal structure indicates that crystal is porous, with characteristic tubular pore structure. Most of these pores run inwards the crystal perpendicularly to external surfaces. The presence of such pores may suggest possible interaction of struvite stone with urine. Urine passing through and outside the crystals may enhance the absorption of other chemicals that can stimulate growth or aggregation of crystals. It can be also assumed that porosity of crystals facilitates bacterial adhesion and biofilm development, in which bacteria surrounded by exopolysaccharides are resistant to antibiotics and host immune defense mechanisms. This phenomenon may explain the rapid growth of infectious urinary stones, persistence and recurrence of this disease.

In addition, material porosity deteriorates its mechanical properties. This issue maybe very important in the case of applying the extracorporeal shock wave lithotripsy, ESWL, which is the most common urological procedure used to remove stones from the urinary tract. The porosity of material is correlated with a tendency to either ductility or brittleness and, consequently, may change the manner by which urinary stones interact with the mechanical stresses produced by ESWL. This is discussed in Ref. [21]. Moreover, the porosity is in agreement with the observations of natural struvite isolated from real urinary stones which are described as porous [19, 22]. However, the internal structure does not confirm good 3D arrangement of small subunits, nanoparticles or microparticles, as it might be suggested by surface pattern. Therefore, the obtained results do not confirm preliminary suggestion [10, 11], that struvite is mesocrystal. The conclusion from the current analysis is that the struvite crystals demonstrate characteristic pattern, which is surface pattern only.

### Struvite morphology and habit

The morphology of crystals forming urinary stones is very important for several reasons. First of all, the morphology of crystals influences the sensitivity to the ESWL, reflects the rate of crystallization and suggests the mechanism of stone formation [20]. Moreover, the appearance of a particular morphology may be a significant indicator of a given disease, as the morphology is different for various diseases [19]. Therefore, in this subsection we focus in more detail on struvite morphology.

**Fig. 6:**
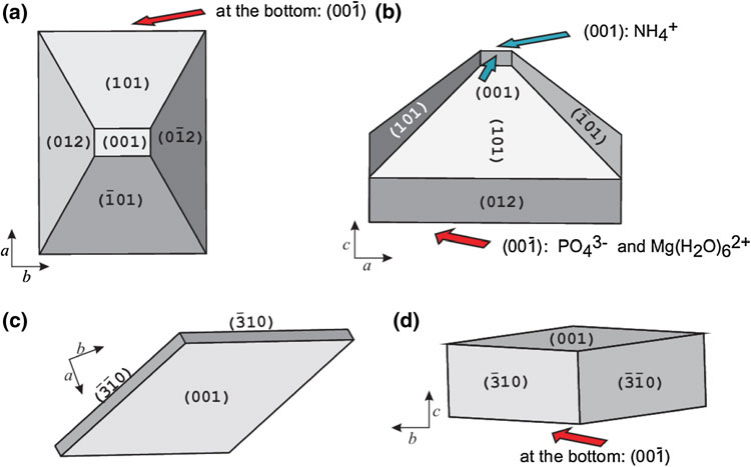
Schematic representation of struvite morphology and habits characteristic for growth from artificial urine with the presence of *P. mirabilis* at relatively low pH value (**a**, **b**) and high value of pH, approximately equal to 9.5 (**c**, **d**). The crystals of the morphology in **c** and **d** are subunits of larger dendritic structures shown in Fig. 2, *middle* and *right columns*. This morphology was drawn on the basis of experimentally observed faces and the (*hkl*) indices are suggested based on these observations and angular measurements only

As we stated earlier, the morphology of struvite crystals and growth process strongly depend on pH value. Single crystals, which are characteristic for low pH, are of coffin-like habit. The most basic crystal morphology is composed of the following faces: (001), $(00\bar{1})$, {101}, $\{ 10\bar{1}\}$, {011}, {012}, and {010} [14]. Usually the $(00\bar{1})$ face is larger than the opposite (001) face (Fig. 1, left column; Fig. 6a, b). The effect of different extension of the (001) and $(00\bar{1})$ faces is due to their different growth rates which are related to their different molecular structure. As described by Abbona, Calleri and Ivaldi [15] the NH_4_^+^ groups terminate the (001) surface, while the groups PO_4_^3−^ and Mg(H_2_O)_6_^2+^ terminate the $(00\bar{1})$ surface (Fig. 6b). Therefore, it is possible that the growth of the $(00\bar{1})$ surface is slowed down by binding the ions, which outcrop this surface by ions present in the solution of urine or the ions related to bacteria existence. This may happen because the surface of bacterial cells usually demonstrates anionic character and therefore they have ability to trap positive ions from the surrounding environment [23]. In the case of *P. mirabilis*, it is shown that capsular polysaccharide and lipopolysaccharide of these microorganisms have the ability to bind the Ca^2+^ and Mg^2+^ ions [24, 25]. Strictly speaking, the polysaccharides which represent the outermost structures of *P.mirabilis* cell show such ability. On this basis, we speculate that the structure and anionic nature of *P. mirabilis* polysaccharides slow down the growth of the $(00\bar{1})$ surface by binding the Mg^2+^ ions which terminate this surface. The growth of the (001) surface is not slowed down and therefore it grows freely, and consequently it is much smaller in comparison with the $(00\bar{1})$ surface. As a consequence, the crystal takes coffin-like habit with large $(00\bar{1})$ surface at the bottom and small (001) surface at the lid. In the case of absence of bacteria, the growth rate of the $(00\bar{1})$ surface may be slowed down by binding other ions present in the urine. However, in this case the crystals of struvite, besides coffin-like habits, display other habits as illustrated in Fig. 3b–f. Therefore, in this case, it seems that such a binding of ions happens rarely.

As we stated earlier the changes in pH value induced transformations in the crystal morphology and habit. At high pH value, twins and dendrite-like structures appear as illustrated in Fig. 1 middle and right columns, respectively. These dendrites are also porous with very characteristic surface pattern (Fig. 2, panels M3 and R4) as discussed in the previous subsection. However, the experimental observations imply that dendrite-like structures correspond to the repetition in three dimensions of the same unit of rhombohedral habit (Fig. 2, panels M1, M2), not coffin-like. This suggests that at high pH (high supersaturation) the habit of primary crystals is modified. It seems that at high pH, the morphology is composed by rhombohedral column such as {310} (Fig. 6c) and probably by the (001) and $(00\bar{1})$ faces. The exact morphology with Miller indices of this type of crystal is unclear. The morphology and Miller indices presented in Fig. 6c, d are suggested on the basis of experimental observation and angle measurements only. These rhombohedral crystals are well aligned with respect to the next crystal and arranged layer-on-layer forming the dendritic structure of X-shaped or with the trunk distinguished. It is possible that this modification in primary crystal is also bacteria-mediated. However, it also possible that in such a structuration the polar character of *c* axis of struvite plays important role. The (001) surface is rich in NH_4_^+^ groups, while the $(00\bar{1})$ surface is rich in the PO_4_^3−^ and Mg(H_2_O)_6_^2+^ groups, so *c* axis is dipole axis. Therefore, it is suggested that the structuration is due to strong dipole–dipole interaction.

To develop and fully explain biological and physical mechanisms of struvite structuration and morphology and habit modification, the growth process and bacteria should be considered as complex dynamical system. The first stage of the understanding these mechanism is to determine which components (proteins, polysaccharides, metabolites) of the system interact. This is a goal of our present research in this field.

## Conclusions

Struvite grown from solution of artificial urine in the presence of *P. mirabilis*, at first stage of growth, take hemimorphic coffin-like habit with large $(00\bar{1})$ face and small (001) face. From the comparison of this result with the results of the control experiments without bacteria, it can be concluded that in the case of absence of bacteria, the crystals in most cases take also the coffin-like habits, but the crystals of other habits are also observed. In addition, in the case of absence of bacteria, the number of crystals is increased and so is their size. In the case of presence of bacteria, it is suggested that the anionic character of the surface of *P. mirabilis* cells and their ability to bind Mg^2+^ ions slow down the growth of the $(00\bar{1})$ face by binding the Mg^2+^ ions which outcrop this face. Consequently, this face increases in size, which leads to different extension of the (001) and $(00\bar{1})$ faces. This result shows a possible complex relationship between nature of bacterial cells and metal binding.

At higher pH, single crystals are observed rarely, and instead twins and dendritic structures appear. Primary crystals of dendritic structures are not coffin-like, but they are rhombohedral. This suggests that at higher pH, the habit of primary crystals is modified. The mechanism of such changes is not clearly understood. However, it seems that polar character of *c* axis of struvite and dipole–dipole interaction may play an important role in this modification.

Single crystals as well twins and dendritic structures show characteristic structuration, which turns out to be only surface pattern. The results do not confirm that the struvite is mesocrystal. However, the internal structure shows porous nature with characteristic tubular pores. The presence of such pores may suggest possible interaction of struvite stone with urine. In particular, the porosity may facilitate the adhesion of bacteria and biofilm development in which bacteria are resistant to antibiotic therapy and immune defense mechanisms.
